# Reduced Graphene Oxide and Polyaniline Nanofibers Nanocomposite for the Development of an Amperometric Glucose Biosensor

**DOI:** 10.3390/s21030948

**Published:** 2021-02-01

**Authors:** Anton Popov, Ruta Aukstakojyte, Justina Gaidukevic, Viktorija Lisyte, Asta Kausaite-Minkstimiene, Jurgis Barkauskas, Almira Ramanaviciene

**Affiliations:** 1 NanoTechnas—Center of Nanotechnology and Materials Science, Institute of Chemistry, Faculty of Chemistry and Geosciences, Vilnius University, Naugarduko st. 24, LT-03225 Vilnius, Lithuania; anton.popov@chgf.vu.lt (A.P.); viktorija.lisyte@chgf.stud.vu.lt (V.L.); asta.kausaite@chf.vu.lt (A.K.-M.); 2 Department of Immunology, State Research Institute Centre for Innovative Medicine, Santariskiu 5, LT-08406 Vilnius, Lithuania; 3 Institute of Chemistry, Faculty of Chemistry and Geosciences, Vilnius University, Naugarduko str. 24, LT-03225 Vilnius, Lithuania; ruta.aukstakojyte@chgf.vu.lt (R.A.); justina.gaidukevic@chf.vu.lt (J.G.); jurgis.barkauskas@chf.vu.lt (J.B.)

**Keywords:** reduced graphene oxide, polyaniline nanostructures, electrochemical glucose biosensor

## Abstract

The control of glucose concentration is a crucial factor in clinical diagnosis and the food industry. Electrochemical biosensors based on reduced graphene oxide (rGO) and conducting polymers have a high potential for practical application. A novel thermal reduction protocol of graphene oxide (GO) in the presence of malonic acid was applied for the synthesis of rGO. The rGO was characterized by scanning electron microscopy, X-ray diffraction analysis, Fourier-transform infrared spectroscopy, and Raman spectroscopy. rGO in combination with polyaniline (PANI), Nafion, and glucose oxidase (GOx) was used to develop an amperometric glucose biosensor. A graphite rod (GR) electrode premodified with a dispersion of PANI nanostructures and rGO, Nafion, and GOx was proposed as the working electrode of the biosensor. The optimal ratio of PANI and rGO in the dispersion used as a matrix for GOx immobilization was equal to 1:10. The developed glucose biosensor was characterized by a wide linear range (from 0.5 to 50 mM), low limit of detection (0.089 mM), good selectivity, reproducibility, and stability. Therefore, the developed biosensor is suitable for glucose determination in human serum. The PANI nanostructure and rGO dispersion is a promising material for the construction of electrochemical glucose biosensors.

## 1. Introduction

It is expected that the global market of medical devices will reach $342.9 billion by 2021 [1]. The market of electrochemical glucose biosensors is one of the fastest growing and was valued at $12.8 billion in 2019 [2]. Moreover, the estimation of glucose concentration is a critical factor in the production of food and beverage [3], in which the glucose concentration can vary significantly. Under these conditions, the multi-purpose use of biosensors can be achieved by employing electrochemical biosensors with a wide linear range. Notably, electrochemical glucose biosensors in general are cheaper, simpler, and faster in comparison to other methods, such as ultraviolet–visible spectroscopy, high-performance liquid chromatography, mass spectrometry, etc. [4,5].

Graphene is a carbon material with high electrical conductivity, resulting from sp^2^-hybridized carbon atoms with out-of-plane π bonds [6]. Despite unique physical, chemical, mechanical, electrical, and thermal properties, pristine graphene is rarely used for the fabrication of biosensors due to its high cost and lack of manufacturing scalability. Meanwhile, other graphene derivatives, namely graphene oxide (GO) and reduced graphene oxide (rGO), are commonly used as the basis of electrochemical biosensors, optimizing the electron transfer process [7,8]. Nowadays, thermal reduction of GO is attracting reasonable attention compared to chemical, electrochemical, or light-driven reduction techniques as it is a simple, low-cost, fast, green, and high-yield synthesis method for rGO production [9]. However, the relative high annealing temperature (above 800 °C), which is frequently used to reduce GO, results in fragmentation of the rGO structure and the generation of structural disorders such as topological defects and carbon vacancies [10]. Furthermore, the thermal reduction of GO at high temperatures is less controlled because of the rapid decomposition of oxygen-containing functional groups, giving a large volume of CO and CO_2_ gases [11]. The efficient recovery of the conjugated π-electron system of rGO could be achieved by using a lower reduction temperature and a suitable reducing agent. Malonic acid is a non-toxic dicarboxylic acid that decomposes at 135 °C [10]. There is very little literature on the use of malonic acid for GO reduction. The successful synthesis of ultrathin rGO sheets by chemical reduction using malonic acid as an effective reducing agent was reported by Kumar [12]. However, there is no evidence of the synthesis protocols for the malonic-acid-assisted thermal reduction of GO. Thus, it was expected that the addition of malonic acid during the thermal exfoliation of GO could help to achieve a higher degree of GO reduction and more effective reconstruction of a π-conjugated graphene structure, resulting in better physical and electrical properties of the final product. In addition, higher electrical conductivity leads to hydrophobic properties of the rGO surface. Since the structural stability, immobilization efficiency, and substrate binding affinity of glucose oxidase (GOx) depend on the hydrophobicity of the substrate [13], the employment of rGO nanocomposites is a key factor for the maintenance of immobilized GOx activity, keeping the positive effect of rGO on the biosensor analytical signal [14,15].

Polyaniline (PANI) is a conducting polymer with unique physical properties [16,17,18]. PANI nanostructures are being increasingly employed as a matrix for the immobilization of enzymes such as GOx, ensuring their activity and stability [19,20]. Three-dimensional porous structures of PANI nanofibers films are especially suitable for enzyme immobilization [21]. Such structures could be formed by controlling the dimensions of PANI nanofibers by changing the conditions of synthesis [22]. For instance, the interfacial polymerization method could be applied. Using it, PANI nanofibers are readily formed without the need for templates or functional dopants [23]. Doping of PANI with metallic nanoparticles [24], various carbon-based materials [25], etc., is commonly performed to enhance the analytical signal by improving the electrochemical biosensor performance.

A new method of thermal reduction of GO in the presence of malonic acid was developed for the production of rGO. The applicability of the rGO and PANI nanostructure dispersion as a matrix for GOx immobilization in the design of a glucose biosensor was evaluated. To achieve the best performance of the biosensor, the PANI and rGO ratio in the dispersion used for the premodification of a graphite rod (GR) working electrode was optimized by evaluating the analytical parameters of the glucose biosensor.

## 2. Materials and Methods

### 2.1. Synthesis of PANI Nanostructures

Polyaniline nanostructures were synthesized using interfacial polymerization according to the protocol reported by Abdolahi et al. [26]. Aniline (Fluka, Neu-Ulm, Germany) was distilled prior to use. Two solutions were prepared as follows: 20 mL of 1 M HCl (Carl Roth, Karlsruhe, Germany) aqueous solution with 4 mM of ammonium persulfate (Fluka, Neu-Ulm, Germany) and 20 mL of 4 mM of aniline solution in chloroform (Penta Praha, Czech Republic). Solutions were stirred separately for 1 h before synthesis. The aqueous solution was further slowly added above the organic phase. A reaction occurred for 24 h. After polymerization, the aqueous phase was collected and the green PANI precipitate was filtered and washed, first with 0.1 M HCl (Sigma-Aldrich, Steinheim, Germany) and then with i-propanol (Sigma-Aldrich, Steinheim, Germany), to remove impurities such as the oxidizing agent, oligomers, and the residual monomer. Synthesized PANI nanostructures were dried at room temperature.

### 2.2. Synthesis of GO

GO was prepared according to the protocol described in the work of Yan et al. [27]. In a typical synthesis, graphite powder (Merck, Darmstadt, Germany) (6.0 g) was treated with K_2_S_2_O_8_ (Sigma-Aldrich, Steinheim, Germany) (5.0 g) and P_2_O_5_ (Carl Roth, Karlsruhe, Germany) (5.0 g) in 98 wt% sulfuric acid (Carl Roth, Karlsruhe, Germany) (24.0 mL) medium. Then, the dry, pre-oxidized graphite was subjected to oxidation, following the methodology reported by Hummers et al. [28]. GO produced in this way was filtered and washed with 0.5 L of 10% HCl aqueous solution to remove metal ions. Afterward, the GO particles were suspended in deionized water and purified using a dialysis tubing cellulose membrane with a cutoff molecular weight of 10,000–20,000 Da (Carl Roth, Karlsruhe, Germany) for 14 days until the pH value of the dialysate became ~5. The resulting GO suspension was filtered using a Buchner funnel, and the obtained brown powder was dried under ambient atmosphere to a constant weight.

### 2.3. Thermal Reduction of GO

GO and malonic acid (Sigma-Aldrich, Steinheim, Germany) in a weight ratio of 1:3 were ground in an agate mortar for 10 min. Afterward, the prepared solid mixture was annealed in a horizontal tube furnace (SNOL, Utena, Lithuania) under argon (Elme Messer Gaas, Vilnius, Lithuania) flow (60 mL min^−1^) at 600 °C for 30 min. Finally, the thermally reduced mixture of GO and malonic acid was washed with deionized water and dried in air. The synthesized black product was denoted as rGO.

### 2.4. Material Characterization

The shapes and morphologies of the synthesized materials were analyzed using a scanning electron microscope (SEM) (SU-70; Hitachi, Tokyo, Japan) at magnifications of 20,000 and 50,000.

The elemental composition and purity were confirmed by energy-dispersive X-ray (EDX) analysis by scanning electron microscopy (TM3000; Hitachi, Tokyo, Japan) equipped with an EDX detector.

X-ray diffraction (XRD) analysis was performed using a MiniFlex II diffractometer (Rigaku, Tokyo, Japan). The diffractograms were recorded in the 2*θ* range from 5° to 60° using CuK_α_ *λ* = 1.5406 Å radiation. Measurements were carried out at 30 kV and 15 mA with a step size of 0.010° and a dwell time of 1.0 s. The interlayer distance *d* for graphite, GO, and rGO was evaluated using Bragg’s equation,

*nλ = 2dsinϴ,* (1)

where *n* is a positive integer, *λ* is the wavelength of the X-ray beam, *d* is the distance between the adjacent interlayers, and *θ* is the diffraction angle.

Fourier-transform infrared (FTIR) spectroscopy measurements were obtained by an FTIR spectrometer (PerkinElmer, Inc., Waltham, MA, USA) in the range of 700–4000 cm^−1^. Infrared spectra were recorded at a 4 cm^−1^ resolution and using the KBr pellet technique in transmission mode.

Raman spectra were recorded using an inVia Raman spectrometer (Renishaw, Gloucestershire, UK) equipped with a thermoelectrically cooled (−70 °C) CCD camera. The He-Ne gas laser was provided an excitation beam at 532 nm with power restricted to 1 mW. The integration time was 100 s.

### 2.5. Preparation of PANI and rGO Dispersions

Material dispersions used for further GR electrode modification were prepared in i-propanol prior to use. One milligram of PANI nanostructures was dissolved in 100 μL of i-propanol solution containing 0.5% Nafion (Alfa Aesar, Kandel, Germany). Prior to use, the dispersion was stirred vigorously in an ultrasonic bath for 15 min. The rGO solution was prepared in the same manner as the PANI dispersion. Then, the PANI and rGO dispersions were mixed and added to the ultrasonic bath for an additional 15 min to prepare PANI and rGO mixtures of the required (5:1, 1:1, 1:5, 1:10, and 1:15) ratios.

### 2.6. Pre-treatment of the Electrode

GR (Sigma-Aldrich, St. Louis, MO, USA) with a working surface area of 0.71 cm^2^ was cut into 4 cm length pieces. The working surface of each electrode was alternately hand-polished with different types (P120, P320, and P2000) of emery paper, washed with distilled water, and dried at room temperature. The lateral surface of the GR electrodes was isolated with a silicone tube to prevent contact with the solution.

### 2.7. Electrode Modification

GOx (Carl Roth, Karlsruhe, Germany) from *Aspergillus niger* (EC.1.1.3.4.) of 360 U mg^−1^ enzymatic activity was used for biosensor design. Four different types of GR electrodes were prepared: modified just with GOx (GR/GOx), modified with PANI nanostructures and GOx (GR/PANI/GOx), modified with rGO and GOx (GR/rGO/GOx), and modified with a PANI and rGO mixture and GOx (GR/PANI:rGO/GOx). Shortly, 6 μL of the required dispersion was deposited on the electrode surface. After evaporation of the solvent, 3 μL of 40 mg mL^−1^ GOx solution was added on the surface and the evaporation procedure was repeated. The modified electrode was hung over a 25% glutaraldehyde (Carl Roth, Karlsruhe, Germany) solution for 15 min and rinsed with distilled water.

Forty milligrams per milliliter of GOx and 1 M of D-(+)-glucose stock solutions were prepared in an acetate-phosphate-buffered saline (A-PBS) solution (pH 6.0) containing 5 mM of CH_3_COONa (AppliChem, Darmstadt, Germany), 5 mM of Na_2_HPO_4_ × 12 H_2_O (Carl Roth, Karlsruhe, Germany), 5 mM of NaH_2_PO_4_ × H_2_O (AppliChem, Darmstadt, Germany), and 0.1 M of KCl (Scharlau, Sentiment, Spain). The glucose solution was kept for 24 h before use in order to reach an equilibrium between glucose isomers.

### 2.8. Amperometric Evaluation of the Biosensor

**Figure 1:**
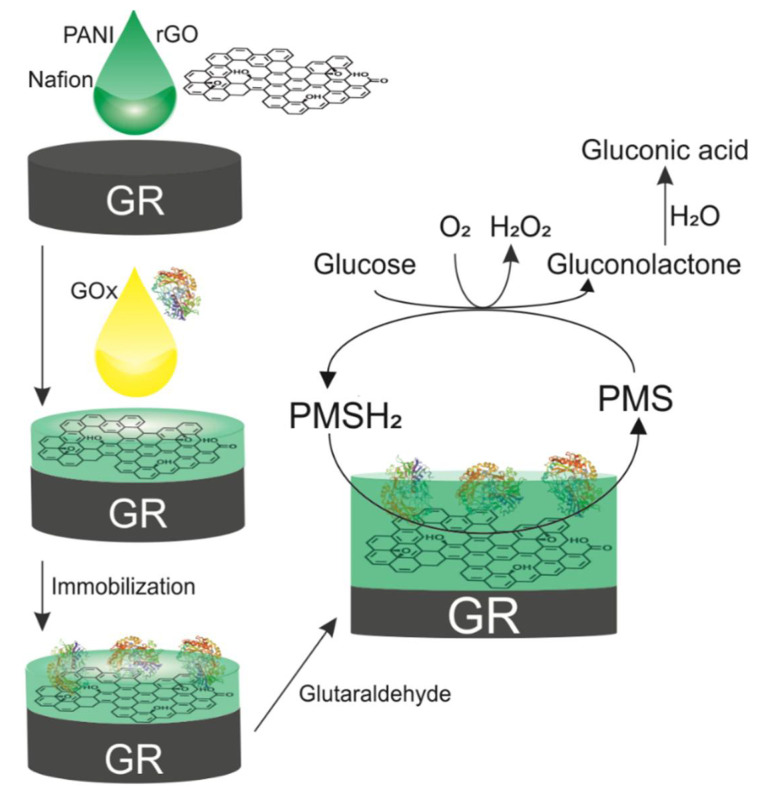
Schematic representation of working GR/PANI:rGO/GOx electrode development and the reaction mechanism of the fabricated biosensor.

Amperometric measurements were performed with potentiostat/galvanostat Autolab PGSTAT 30 (Eco Chemie, Utrecht, The Netherlands) with NOVA software using a three-electrode system consisting of a modified GR electrode, which was employed as a working electrode; an Ag/AgCl _3 M KCl_ electrode (Metrohm, Herisau, Switzerland) as a reference electrode; and a platinum wire as an auxiliary electrode. All experiments were performed at a constant +0.3 V potential in A-PBS solution (pH 6.0) with 6 mM of *N*-methylphenazonium methyl sulfate (PMS) (Sigma-Aldrich, St. Louis, MO, USA). The solution was mixed with a magnetic stirrer at 300 rpm during anodic current response measurements. After a steady current was reached (stable baseline), a corresponding amount of glucose was added into the electrochemical cell. The anodic current change (*ΔI*) after addition of glucose was monitored as an analytical signal of the developed biosensor. During GOx-catalyzed oxidation of glucose, the transfer of electrons from the enzyme redox center to the electrode occurs via the PMS present in the solution (Figure 1). Additionally, PANI or rGO can facilitate electron transfer. The electrochemical results were shown as a mean value of three independent experiments.

### 2.9. Calculations

Michaelis–Menten kinetics was used for the evaluation of the biocatalytic performance of immobilized GOx. The obtained results were approximated using the hyperbolic function *y* = *ax/(b* + *x*). The maximal current (Δ*I_max_*) generated under saturated substrate conditions was the *a* parameter, while the apparent Michaelis constant (*K_Mapp._*) was the *b* parameter of the hyperbolic function.

OriginPro software (version 2017) was used for the Δ*I_max_* and *K_Mapp._* calculation. Calibration curve parameters, such as slope and correlation coefficient, were evaluated. The limit of detection (LOD) as the lowest concentration of the analyte that gives an analytical signal greater than the background value plus 3*δ* was also estimated using the statistic software OriginPro.

## 3. Results and Discussion

**Figure 2:**
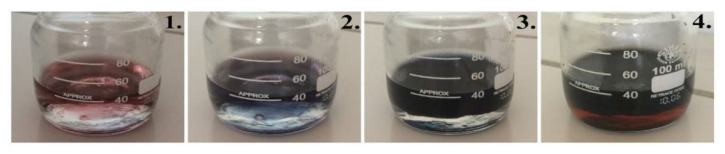
Photographs of the polymerization solution taken after (**1**) 5 s, (**2**) 1 min, (**3**) 1 h, and (**4**) 24 h from the start of the reaction.

**Figure 3:**
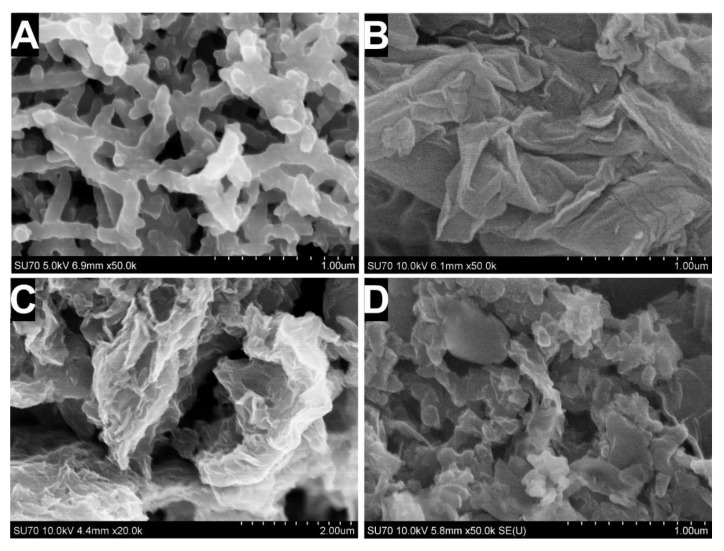
Scanning electron microscope (SEM) images of PANI nanostructures (**A**), GO (**B**), rGO (**C**), and PANI:rGO_1:10_ composite (**D**).

**Figure 4:**
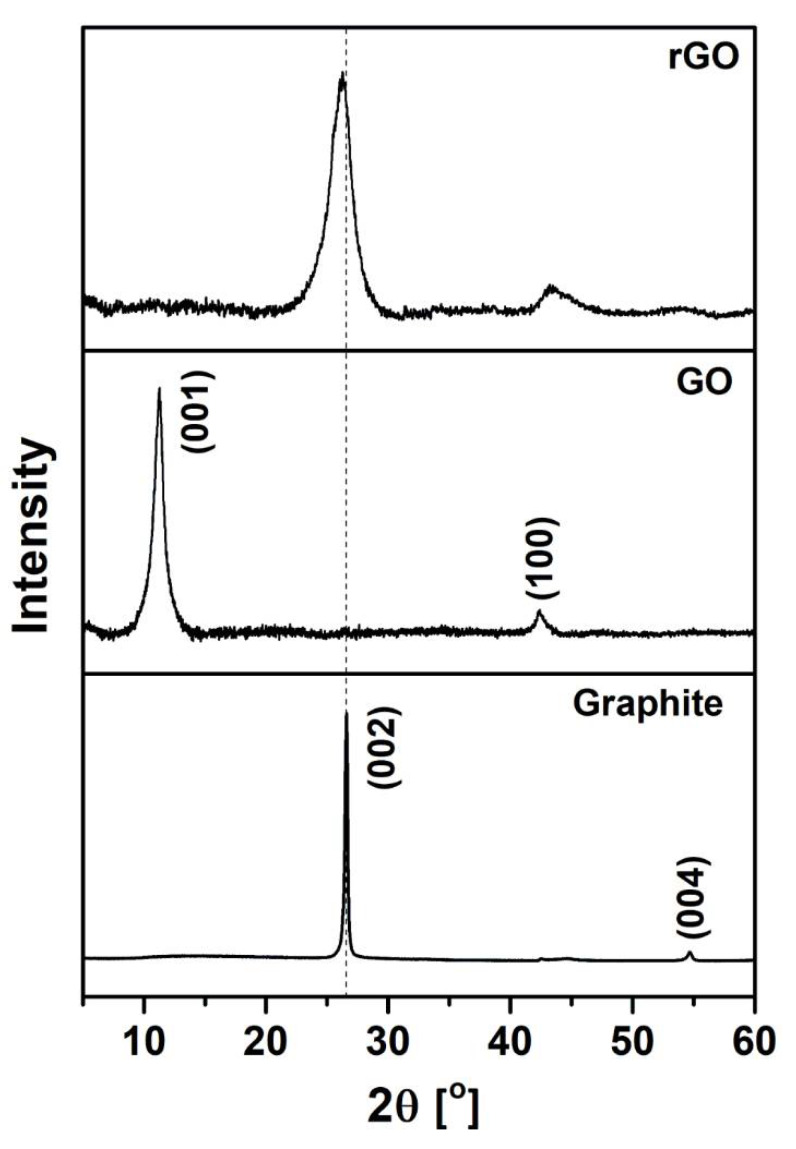
X-ray diffraction (XRD) patterns of graphite, GO, and rGO.

During interfacial polymerization, PANI nanostructures are formed at the interface of two immiscible solutions. After the induction period, which normally takes up to 30 s, PANI nanostructures migrate to the aqueous phase, which is above the interface (Figure 2-1,2). The monitored blue color after the induction stage (Figure 2-2) is connected with the formation of pernigraniline (fully oxidized PANI form) chains, which are further reduced to emeraldine salt [29]. During the synthesis process, accumulation of PANI nanostructures occurs in the aqueous phase, with the color becoming dark green from transparent and turning to black over time due to increased PANI concentration (Figure 2-3,4). A dark-green color indicates the formation of emeraldine salt, which possesses high conductivity among PANI oxidation states. Differences in solubility between aniline, its oligomers, and long PANI chains allow controlling the purity of the prepared PANI nanostructures. The decrease in the number of aniline molecules and polymerization reaction intermediates in the final sample increases the biocompatibility of PANI [30].

The morphology of the synthesized materials was evaluated using the SEM imaging technique (Figure 3). Samples for SEM imaging were prepared by deposition of PANI, GO, and dispersions on GR electrodes. Additionally, a sample of the PANI and rGO dispersion mixed in a ratio equal to 1:10 was prepared. In the case of the PANI sample, nanofibers interconnected to a network-like structure were observed. The diameter of the prepared PANI nanofibers was 118 ± 19 nm, wherein the length varied significantly. A porous slit-shaped structure of the PANI nanofiber network could be used as a matrix for enzyme immobilization, wherein the diameter and length of the PANI fiber can be controlled by changing the concentrations of aniline and ammonium persulfate [26,31].

It is clear from the micrograph illustrated in Figure 3B that the GO sheets were in the form of thin, flexible, and wrinkled layers with multiple folds. The oxidation process promoting the attachment of hydroxyl and epoxy functional groups onto the graphene basal plane could explain this wrinkled aspect. rGO thermally reduced in the presence of malonic acid (Figure 3C,D) was characterized by irregular stacking of graphene layers that were torn, leading to the formation of veil-like sheets with scrolling edges. Moreover, rGO sheets were well exfoliated and possessed a slit-shaped porous structure, which should be more suitable for the preparation of a PANI and rGO nanocomposite in comparison to the layered, wrinkled wave structure intrinsic to graphene [32]. Distinct changes in the morphology of the PANI:rGO_1:10_ nanocomposite (Figure 3D) in comparison to PANI and rGO were obtained. Additionally, PANI nanofibers were distributed randomly on the surface and/or between the rGO sheets.

The obtained results of elemental analysis are listed in Table 1. EDX data of GO reveal the presence of carbon, oxygen, and sulfur at 65.59, 33.57, and 0.84 at%, respectively. The sulfur content is probably associated with residuals of HSO_4_^−^, SO_4_^2−^, and SO_3_^2−^ as a consequence of the graphite intercalation process. In addition, EDX analysis results show that rGO contains 93.94 and 6.06 at% of carbon and oxygen, respectively. It was observed that the C/O ratio for rGO (15.50) is almost eight times higher than for GO (1.95), indicating effective thermal reduction of GO. The elements C, S, N, and Cl were identified in PANI. The presence of Cl and S displays the successful attachment of the doping species onto the polymer. EXD results of PANI:rGO_1:10_ show 93.27, 5.56, 1.04, and 0.13 at% of carbon, oxygen, nitrogen, and chlorine, respectively. These data confirm the successful formation of a PANI:rGO_1:10_ composite.

The structural changes in GO after annealing were investigated by XRD analysis. The recorded diffraction patterns of graphite, GO, and rGO are presented in Figure 4. The diffractogram of pristine graphite shows characteristic peaks at 2*θ* = 26.60° and 2*θ* = 54.70°, which correspond to the planes (002) and (004) of a hexagonal lattice, respectively. According to Bragg’s equation (Equation (1)), the interlayer spacing of the (002) plane reflection equals 0.335 nm, which is a representative d-spacing of stacked graphene sheets in graphite. The diffraction pattern of GO exhibits two peaks at 2*θ* = 11.28° and 2*θ* = 42.38°, corresponding to the reflections of (001) and (100), respectively. The XRD pattern of GO is similar to the XRD data that have been reported by Bandara [33]. The interplanar spacing d_001_ of GO was expanded to 0.784 nm due to the presence of oxygen-containing functional groups between the graphene layers. It reveals the successful oxidation of graphite. The XRD results of the rGO sample show that during the thermal reduction of GO with an additive, the (001) peak disappears, while the (002) peak shifts to the position typical for graphite (2*θ* = 26.33°), with an interlayer distance of 0.338 nm. This can be attributed to the removal of water and oxygen-containing groups and a partial restoration of the sp^2^ carbon network. Furthermore, after the thermal reduction of GO, the peaks in the XRD patterns become broader. This results from a decrease in the crystallite size. Moreover, the diffraction peaks centered at approximately 2*θ* = 26.33° are asymmetric because of the mixed polycrystalline structure of graphitic and disordered domains. The presence of the (002) and (100) planes confirms that the structure of rGO is between the structures of graphite and GO.

**Figure 5:**
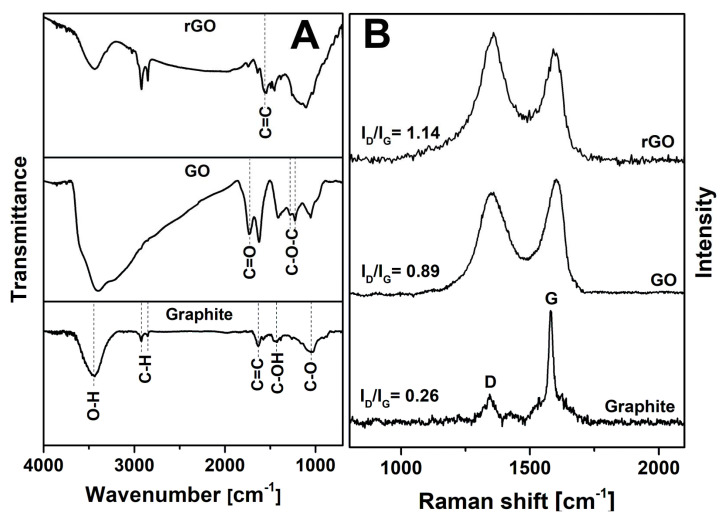
FTIR (**A**) and Raman (**B**) spectra of graphite, GO, and rGO.

Oxygen-containing functional groups of the synthesized products were identified by FTIR spectroscopy. FTIR spectra of graphite, GO, and rGO are compared in Figure 5A. In the FTIR spectrum of graphite, the absorption peaks at 2924 and 2853 cm^−1^ are attributed to the symmetric and asymmetric stretching modes of C–H, which are characteristic of –CH_2_– and –CH_3_ groups [34]. The broad band at 3444 cm^−1^ indicates the vibrations of hydroxyl groups or intercalated water molecules. The peak at 1633 cm^−1^ corresponds to the skeletal vibrations of C=C bonds [35]. The absorption peaks at 1420 and 1052 cm^−1^ may be related to the stretching modes of the tertiary alcohol (C–OH) and phenol (C–O) groups, respectively [36]. The FTIR spectrum of GO shows an abundant number of oxygen-containing groups in the sample. The spectrum of GO is consistent with that presented in literature data [37]. The profile and intensity of the peak observed around 3400 cm^−1^ are changed due to the attachment of hydroxyl groups to the graphene sheets. The new band at 1730 cm^−1^ is associated with the stretching mode of C=O corresponding to the carboxyl group. Moreover, the existence of epoxy groups (C–O–C) was confirmed from the absorption bands in the range of 1300–1200 cm^−1^ [36]. After the thermal reduction, a significant decrease in the intensity of absorption bands related to oxygen-containing groups (C–OH, C=O, and C–O–C) was observed. However, some residual oxygen-containing species remain in the material. In fact, the appearance of the new peak at 1550 cm^−1^ indicates the C=C vibrations attributed to the aromatic carbon rings [38]. This can support the hypothesis that during thermal reduction, the partial restoration of the π-conjugated sp^2^ structure should enhance the electrical conductivity of rGO [39]. The higher intensities of the absorption peaks at 2924 and 2853 cm^−1^ show methylene and ethylene groups on the basal plane and edges of graphene, describing the defective parts of the structure, such as sp^3^ defects, C vacancies, and dislocations. The improved electrical conductivity and more chemically reactive defective sites in the graphene layer make rGO a favored graphene material for the preparation of electrodes for biosensors [40].

Raman spectroscopy is a fast and nondestructive characterization method that gives useful information about the lattice structure, structural disorders, crystallization, and defects of carbon materials [41]. Recorded Raman spectra of graphite, GO, and rGO are shown in Figure 5B. The obtained spectrum of graphite displays two typical bands associated with graphene-based materials. The G band located at 1344 cm^−1^ arises due to the vibrations of sp^2^-hybridized carbon atoms. The mode appearing at 1580 cm^−1^ is the D band, which is related to the structural defects, such as grain boundaries, C vacancies, sp^3^ defects, and armchair and zigzag edges of the carbon material [42]. Its intensity is always low in graphite and high-quality graphene. The G band in GO is shifted to higher wavenumbers (1602 cm^−1^) because of oxygen-containing functional groups and sp^3^ carbon atoms in the structure. The G mode in the spectrum of rGO is shifted to a lower wavenumber (1591 cm^−1^) due to the reduction of graphene oxide, which results in the partial restoration of the π-conjugated carbon network. The intensity ratio of D and G bands (*I*_D_/*I*_G_) could be used to investigate the degree of disorder in the graphene structure [36,41]. The calculated value of *I*_D_/*I*_G_ is 0.26 for graphite. It is noticed that the intensity ratio increases to 0.89 and 1.14 for GO and rGO, respectively. The high *I*_D_/*I*_G_ ratio in GO confirms that its lattice is distorted and has a large number of sp^3^-like defects caused by the oxidation process. Thus, the process of oxidation and reduction leads to an increase in structural defects in the products due to the formation of sp^2^ carbon domains in samples. The broader D and G bands of GO and rGO reveal the lower crystallinity of the samples, which confirms XRD data [43].

**Figure 6:**
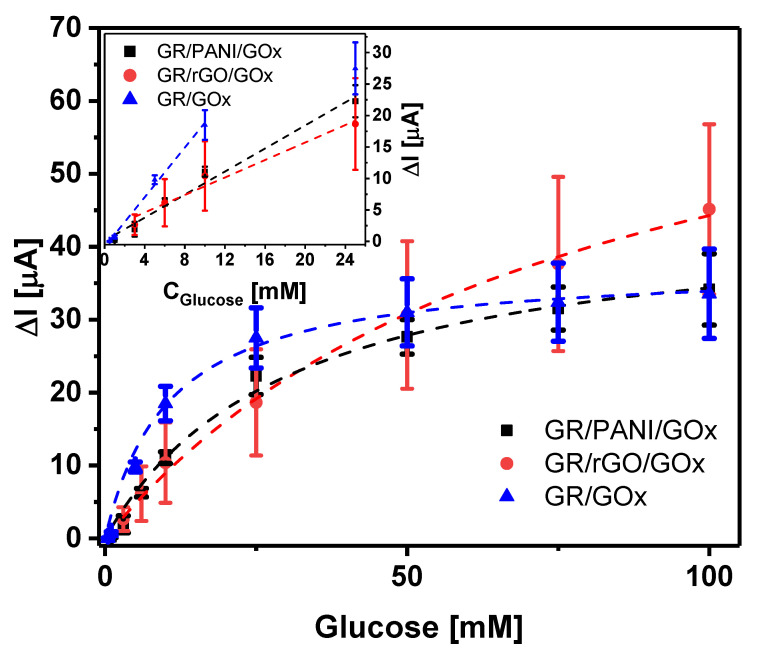
Calibration plots of biosensors based on GR/PANI/GOx, GR/rGO/GOx, and GR/GOx working electrodes. Inset: linear ranges of detection. Conditions: applied potential +0.3 V; acetate-phosphate-buffered saline (A-PBS) solution (pH 6.0) with 6 mM of *N*-methylphenazonium methyl sulfate (PMS).

The dispersions of PANI and rGO in i-propanol containing 0.5% of Nafion were used for the fabrication of GR/PANI/GOx and GR/rGO/GOx electrodes. GOx was adsorbed on the premodified working GR electrode and cross-linked by glutaraldehyde vapor. The effect of rGO and PANI dispersions on the amperometric response of biosensors based on GR/PANI/GOx or GR/rGO/GOx electrodes in the presence of glucose was evaluated and compared with the response of biosensors based on GR/GOx electrodes. The calibration plots are presented in Figure 6.

It is obvious that premodification of the electrodes with a PANI or rGO dispersion affected the electrochemical performance of the fabricated biosensors. Due to the employment of a PANI or rGO dispersion in the construction of the biosensor, the sensitivity decreased from 2.0 μA mM^−1^ for GR/GOx to 0.92 μA mM^−1^ for GR/PANI/GOx and 0.69 μA mM^−1^ for GR/rGO/GOx. However, the linear range of detection for biosensors based both on PANI- and rGO-premodified electrodes became wider. Biosensors based on GR/GOx electrodes had a linear range of detection from 0.5 to 10 mM (*R* = 0.9980), and the linear ranges of detection in the case of GR/PANI/GOx and GR/rGO/GOx electrodes were 1–25 mM (*R* = 0.9908) and 3–25 mM (*R* = 0.9839), respectively. Premodification of the electrodes with PANI nanostructures and rGO also led to an increase in the *K_Mapp_*_._ value from 12.0 ± 1.9 mM (GR/GOx) to 77.7 ± 9.5 mM and 30.5 ± 4.9 mM, respectively, which also testifies to a wider linear range of biosensors [44]. A higher Δ*I_max_* value of the biosensor based on a GR/rGO/GOx (78.7 ± 5.2 μA) electrode in comparison to a GR/PANI/GOx electrode (44.8 ± 2.7 μA) could be related to better electron transfer through the matrix. An increase in the standard deviation (SD) in the case of the biosensor based on the GR/rGO/GOx electrode indicated decreased reproducibility, wherein SD values calculated for biosensors based on GR/PANI/GOx and GR/GOx were comparable. Possible explanations may be found in the better suitability of PANI nanostructures for the role of the matrix for enzyme immobilization, taking into account the hydrophobic rGO nature.

**Figure 7:**
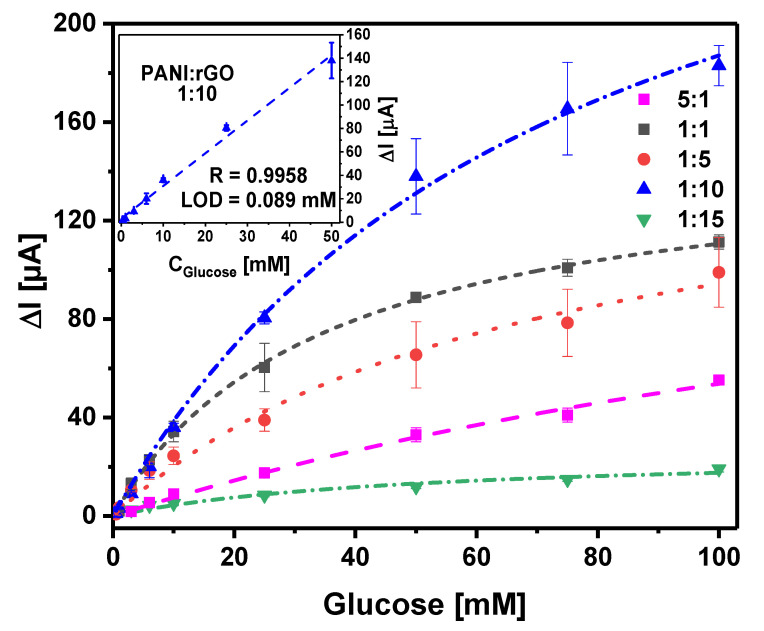
Calibration plots of biosensors based on GR/PANI:rGO/GOx electrodes fabricated using different ratios of PANI and rGO dispersions: 5:1, 1:1, 1:5, 1:10, and 1:15. Inset: the linear range of detection of a biosensor based on a GR/PANI:rGO_1:10_/GOx electrode. Conditions: applied potential +0.3 V; A-PBS solution (pH 6.0) with 6 mM of PMS.

To improve the glucose biosensor’s performance, another premodification of the GR electrode strategy was implemented by using both PANI and rGO dispersions together. The effect of the PANI and rGO dispersion ratio on the analytical signal of the developed biosensors was studied to select the optimal composition of PANI and rGO. The calibration plots are presented in Figure 7.

It was found that a change in the composition of the dispersion used for the premodifcation of the working electrode strongly affects the analytical performance of the biosensor. A biosensor based on an electrode premodified with PANI and rGO dispersions mixed in a ratio equal to 5:1 was characterized by quite a low current response. The sensitivity of the biosensor based on the GR/PANI:rGO**_5:1_**/GOx electrode was equal to 1.0 μA mM^−1^, while the linear range of detection was from 3 to 100 mM. A gradual increase in the rGO amount in the dispersion allowed improving the analytical characteristics of the developed biosensors. It is obvious that the electrode premodified with dispersions of PANI and rGO mixed in a ratio equal to 1:10 showed the best performance. The linear range of detection of the GR/PANI:rGO**_1:10_**/GOx biosensor became wider in comparison with the above-described biosensors (Figure 6), and it was from 0.5 to 50 mM (*R* = 0.9958). The sensitivity of the biosensor based on the GR/PANI:rGO**_1:10_**/GOx electrode was equal to 2.8 μA mM^−1^, and the LOD was estimated to be 0.089 mM. The limit of quantification (LOQ) was calculated as 0.30 mM at a signal-to-noise ratio of 10 standard deviations. The developed biosensor possessed good reproducibility, since the relative standard deviation (RSD) calculated from three independent measurements for 3, 10, and 100 mM of glucose was, respectively, equal to 4.66%, 4.71%, and 4.48%. Moreover, the PANI:rGO dispersion matrix due to the increase in the diffusion limitation had a significant influence on the *K_Mapp_*_._ value. It was 12.0 ±1.9 mM for the biosensor based on the GR/GOx electrode, while for the biosensor based on the GR/PANI:rGO**_1:10_**/GOx electrode, *K_Mapp_*_._ was equal to 74.4 ± 9.0 mM.

It should be noted that a further increase in the PANI and rGO ratio up to 1:15 had a negative impact on biosensor performance. This clearly illustrates that a PANI-to-rGO ratio of 1:10 is optimal for the premodification of the working electrode. This observation suggests that a PANI nanostructure matrix environment encourages the maintenance of enzyme activity during measurements. The hydrophobic rGO nature should not be forgotten in this context. An excessive amount of rGO in the matrix could be the reason for GOx molecules’ conformational alterations affecting the stability and catalytic activity of the enzyme [45]. On the other hand, due to the presence of rGO in the matrix, the electron transfer rate through the matrix was possibly enhanced.

The analytical parameters of the designed biosensor and other glucose biosensors based on GOx are summarized in Table 2. It clearly illustrates that the linear range was wider in the case of biosensors based on GR/PANI:rGO_1:10_/GOx electrodes in comparison to other biosensors intended for glucose concentration detection.

The performance of an amperometric glucose biosensor based on the GR/PANI:rGO_1:10_/GOx electrode was evaluated in human serum diluted 10 times with A-PBS solution. Electrochemical measurements were performed after the addition of 5 mM of glucose to the serum. The obtained results demonstrate good accuracy. The glucose concentration calculated as a mean value of three independent experiments was equal to 4.99 ± 0.11 mM.

**Figure 8:**
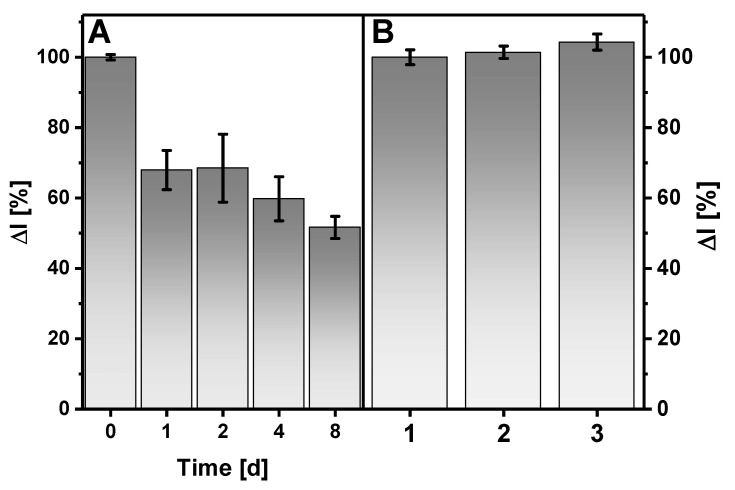
Anodic current response of the biosensor based on a GR/PANI:rGO_1:10_/GOx electrode to glucose vs. time (**A**) and in the absence (**1**) and presence of (**2**) 0.6 mM of uric acid or (**3**) 0.2 mM of ascorbic acid (**B**). Conditions: applied potential +0.3 V; (**A**) A-PBS solution with 6 mM of PMS and 25 mM of glucose; (**B**) human serum diluted 10 times with A-PBS in the presence of 6 mM of PMS and 5 mM of glucose.

Stability is an important parameter of the glucose biosensor, which refers to the precision of the same glucose concentration measurements over a period of time under continuous monitoring. The current response generated after the addition of 25 mM of glucose to A-PBS solution (pH 6.0) with 6 mM of PMS was monitored over an 8-day period (Figure 8A). The fabricated GR/PANI:rGO_1:10_/GOx electrode was kept above A-PBS solution (pH 6.0) in a closed vessel at 4 °C. A significant decrease in the measured current response by 32% was registered after 1 day. However, on the 8th day, the current response was still higher than 50% of the initial analytical signal.

Some electroactive species coexisting in a real sample of human serum could influence the current response of the electrochemical biosensors [36]. The effect of the presence of 0.2 mM of ascorbic acid (AA) and 0.6 mM of uric acid (UA) in the solution on the amperometric response of a biosensor based on a GR/PANI:rGO_1:10_/GOx electrode was studied. Such concentrations were chosen since the typical AA and UA concentrations in the blood serum for a healthy adult are in the range of 0.040–0.141 and 0.149–0.446 mM, respectively [50]. Serum diluted 10 times with A-PBS solution was used to imitate real samples. As can be seen from Figure 8B, the registered analytical signal increased by about 4.3% and 1.4% when ascorbic acid and uric acid were present, respectively, in the solution. The tested concentrations of AA and UA are comparable with the physiological condition. So, it could be assumed that the tested electroactive species insignificantly influence the accuracy of the amperometric response during measurements of glucose concentration in diluted human serum. In the proposed system, a layer of PANI nanostructures and rGO increases the diffusion barrier, thus complicating species transport to the GR electrode surface. The remaining carboxyl groups in the structure of rGO are deprotonated and negatively charged at pH 6.0. Therefore, these functional groups electrostatically repel electroactive interference, which are similarly charged. Moreover, the effect of electroactive species possibly could be reduced using additional cross-linking of GOx with Nafion. This can act as an effective semipermeable barrier [51], which significantly complicates the penetration of anionic interferences.

**Figure 9:**
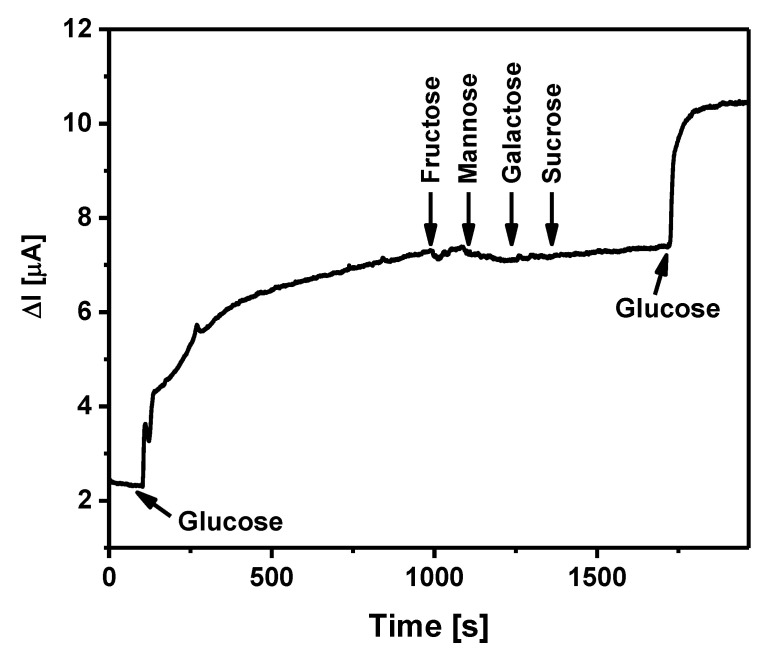
Current response of the biosensor based on a GR/PANI:rGO_1:10_/GOx electrode to glucose, fructose, mannose, galactose, and sucrose. Conditions: applied potential +0.3 V; A-PBS solution with 6 mM of PMS; 5 mM of glucose, followed by addition 5 mM of fructose, 5 mM of mannose, 5 mM of galactose, 5 mM of sucrose, and again 5 mM of glucose.

The selectivity of the developed biosensor was evaluated by adding aliquots of five carbohydrates. In this experiment, 5 mM of glucose, 5 mM of fructose, 5 mM of mannose, 5 mM of galactose, 5 mM of sucrose, and again 5 mM of glucose were added one after the other to the electrochemical cell, while waiting for the reaching a saturation current response after previous sugar addition. It is obvious (Figure 9) that the addition of the sugars (excluding glucose) did not affect the amperometric response of the designed biosensor. These results allow us to state that the biosensor based on a GR/PANI:rGO_1:10_/GOx electrode is selective to glucose in comparison to other tested carbohydrates.

## 4. Conclusions

The study demonstrated the methods that are suitable for the synthesis of rGO thermally reduced at a relative low temperature of 600 °C in the presence of malonic acid. A dispersion of synthesized rGO with PANI nanostructures can be used for the premodification of the working electrode and as a matrix for GOx immobilization, improving the electrochemical performance of the glucose biosensor. Moreover, a PANI and rGO ratio equal to 1:10 was selected and found to be optimal for working electrode premodification and allowed us to achieve the best analytical parameters of the biosensor. The developed biosensor is suitable for determining the glucose concentration in human serum. Additionally, due to the broad linear range of detection, the designed biosensor can be applied to samples with high glucose concentrations or in which the glucose concentration could vary considerably. Moreover, such an analytical system may be appropriate for the determination of the typical glucose concentration in undiluted or diluted blood samples. We believe that while PANI acts as a matrix for enzyme immobilization, the rGO presence in the matrix facilitates electron transfer from the GOx redox center to the electrode in the presence of a soluble redox mediator and increases the current response of the biosensor. Although the premodified working electrode was used for immobilization of GOx and further determination of glucose, it could be easily adapted to other biosensors based on a wide variety of enzymes.

## Figures and Tables

**Table 1:** Energy-dispersive X-ray (EDX) analysis results of synthesized materials.

	Atomic Composition (%)				
	C	O	S	N	Cl
**GO**	65.59	33.57	0.84	-	-
**rGO**	93.94	6.06	-	-	-
**PANI**	93.78	-	0.51	3.39	2.31
**PANI:rGO_1:10_**	93.27	5.56	-	1.04	0.13

**Table 2:** Comparison of different amperometric glucose biosensors.

Glucose Biosensor	Linear Range (mM)	LOD (mM)	*K_Mapp._* (mM)	Reference
**GR/PANI:rGO/GOx**	0.5–50	0.089	74.4	Current work
**SPCE/MWCNTs:AuNP:rGO:/GOx**	1–10	0.064	0.74	[24]
**GR/rGO/PANI/GOx**	0.1–8.5	0.0007	1.52	[25]
**CC/PANI/GOx**	1–8	0.050	-	[46]
**Au/PANI:PAA/GOx**	0.1–1.3	0.024	-	[47]
**GCE/G:GOx**	0.1–10	0.010	-	[48]
**GCE/rGO:C60/GOx**	0.1–12.5	0.035	4.44	[49]

Note: SPCE, screen-printed carbon electrode; MWCNTs multiwall carbon nanotubes; AuNP, gold nanoparticle; CC, carbon cloth; PAA, poly(acrylic acid); GCE, glassy carbon electrode; G, graphene; C60, fullerene-C60.
